# Cyclobrachycoumarin from *Gerbera piloselloides* Inhibits Colorectal Cancer In Vitro and In Vivo

**DOI:** 10.3390/molecules29235678

**Published:** 2024-11-30

**Authors:** Limei Fan, Xiansheng Ye, Qian Fang, Xiaoxuan Li, Haiping Wang, Binlian Sun, Xiji Shu, Xiaoying Hou, Yuchen Liu

**Affiliations:** 1 Cancer Institute, School of Medicine, Jianghan University, Wuhan 430056, China; limei_fan1226@jhun.edu.cn (L.F.); fangq002233@163.com (Q.F.); xiaoxuan2001@yeah.net (X.L.); wang-haiping@jhun.edu.cn (H.W.); binlian17@jhun.edu.cn (B.S.); 2 Hubei Key Laboratory of Cognitive and Affective Disorders, Wuhan Institute of Biomedical Sciences, School of Medicine, Jianghan University, Wuhan 430056, China; yxs2021@jhun.edu.cn (X.Y.); xijishu@jhun.edu.cn (X.S.)

**Keywords:** *Gerbera piloselloides*, cyclobrachycoumarin, colorectal cancer, apoptosis, cell cycle

## Abstract

*Gerbera piloselloides*, a plant in the *Asteraceae* family, is a traditional Chinese medicinal herb known for its unique therapeutic properties, including reported anti-tumor and antioxidant effects. Recent studies suggest that the main constitute of *G. piloselloides*, coumarins, may have potential anti-tumor activity. Recent research suggests that coumarins, the active compounds in *G. piloselloides*, may hold potential anti-tumor activity. However, the pharmacodynamic constituents remain unidentified. This study aims to isolate and characterize the bioactive compounds of *G. piloselloides* and to assess its anti-tumor effects. Initially, seven compounds, including coumarins, a ketone, and a furanolide, were isolated and identified from *G. piloselloides* by semi-preparative high-performance liquid chromatography (HPLC) and nuclear magnetic resonance (NMR) analysis. The anti-tumor effects of these compounds were evaluated across four different cancer cell lines. Among them, the compound cyclobrachycoumarin showed a significant inhibitory effect on colorectal cancer (CRC) cell proliferation and was selected for further investigation. Cyclobrachycoumarin was found to induce CRC cell apoptosis and cell cycle arrest in a dose-dependent manner. This treatment also led to increased levels of ROS and cleaved PARP, along with decreased expressions of survivin, cyclin D1, and CDK1. In vivo studies further demonstrated that cyclobrachycoumarin effectively reduced tumor growth in HT-29 xenograft models by promoting apoptosis and cell cycle arrest, with a favorable tolerability profile. In summary, this study suggests that cyclobrachycoumarin may be a promising candidate for safe and effective CRC therapy.

## 1. Introduction

Cancer is a global public health crisis, with incidence and mortality rates continuing to rise [1]. Colorectal cancer (CRC) is among the most prevalent malignant tumors, characterized by a high tendency for metastasis and poor prognosis [2]. Recent cancer statistics show that CRC ranks among the top five cancers worldwide and in China, in terms of both morbidity and mortality [3,4]. Current CRC treatments include surgical intervention, radiation therapy, and systemic chemotherapy, with chemotherapy as the primary treatment for advanced cases [5,6]. However, prolonged chemotherapy often leads to severe toxicity and drug resistance, ultimately resulting in treatment failure [7,8]. For instance, first-line drugs such as oxaliplatin (OXA) and 5-fluorouracil (5-Fu) are limited by systemic toxicity and resistance [9,10], underscoring the need for safer, more effective therapies.

Traditional Chinese medicine (TCM) offers abundant resources for treating a variety of diseases [11]. Due to their complex, multi-component, and low-toxicity characteristics, bioactive compounds from TCM have the potential to serve as novel, promising medications [12]. *Gerbera piloselloides* (L.) Cass., a member of the *Asteraceae* family, is commonly used as an ornamental plant abroad, but in China, it is valued for its medicinal properties [13]. Historically, *G. piloselloides* has been used to clear heat, relieve cough, and promote lung health [14]. Today, it is applied in treating inflammatory conditions, such as fevers and coughs [15]. Coumarins, the main bioactive components of *Gerbera*, have demonstrated anti-cancer, anti-inflammatory, and antioxidant properties [16,17,18,19]. For example, compounds like gerberchromone D and gerbeloid J extracted from *G. piloselloides* have shown significant inhibitory effects on the breast cancer cell line MDA-MB-231 [20]. Although *G. piloselloides* is widely used in clinical practice, its active compounds remain poorly understood, and its potential anti-tumor applications warrant further investigation.

In our study, seven compounds were isolated from *G. piloselloides*, and their anti-tumor activities were further conducted. First, we compared the anti-proliferative effects of these compounds across four human tumor cell lines to identify the candidate compound. We then assessed anti-cancer activity in CRC cells by examining apoptosis and cell cycle changes. Additionally, we analyzed the intracellular ROS level and performed Western blotting to explore the potential mechanism. Finally, we confirmed the compound’s inhibitory effect in vivo using a xenograft transplantation model. In summary, this study investigated the anti-CRC effect of the bioactive constituent from *G. piloselloides*, highlighting its potential as a safe and effective therapeutic agent for CRC.

## 2. Results

### 2.1. Seven Active Constituents Were Isolated and Identified from G. piloselloides

**Figure 1:**
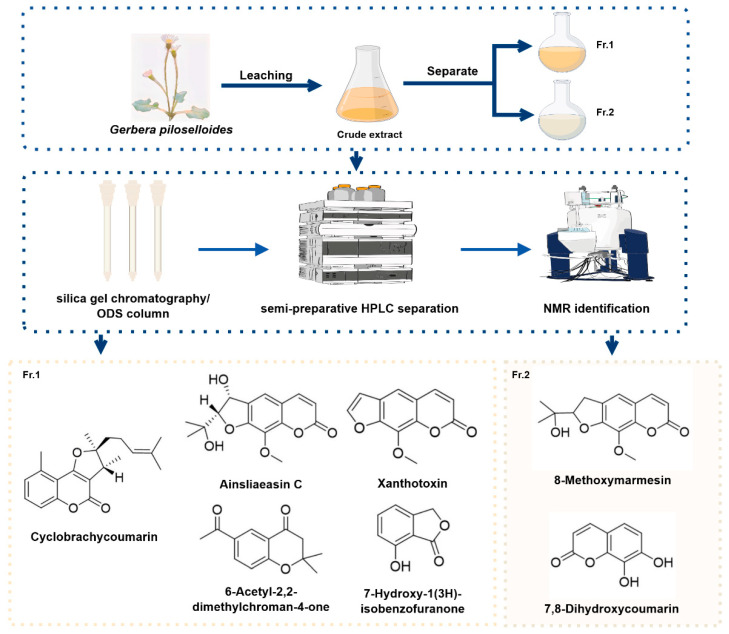
Seven active constituents were isolated and identified from *G. piloselloides*. The crude extract of *G. piloselloides* was first obtained through vacuum extraction, followed by purification using silica gel chromatography, yielding two primary fractions: Fr.1 and Fr.2. These fractions were further separated using multiple column chromatography techniques and semi-preparative HPLC, ultimately isolating seven compounds. The structures of the isolated compounds were identified as follows: cyclobrachycoumarin, ainsliaeasin C, 8-methoxymarmesin, xanthotoxin, 7,8-dihydroxycoumarin, 6-acetyl-2,2-dimethylchroman-4-one, 7-hydroxy-1(3H)-isobenzofuranone.

In the search for active constituents, ethanol extraction, followed by silica gel chromatography, ODS column chromatography, and semi-preparative HPLC, was used, leading to the isolation of seven compounds. Based on previous studies and comprehensive NMR data analysis, their structures were identified as cyclobrachycoumarin [21], ainsliaeasin C [22], 8-methoxymarmesin [23], xanthotoxin [24], 7,8-dihydroxycoumarin [25], 6-acetyl-2,2-dimethylchroman-4-one [26]^,^ and 7-hydroxy-1(3H)-isobenzofuranone [17] (Figure 1 and Figures S1–S14). Among these compounds, cyclobrachycoumarin, ainsliaeasin C, 8-methoxymarmesin, xanthotoxin, and 7,8-dihydroxycoumarinwere classified as coumarins, 6-acetyl-2,2-dimethylchroman-4- as a ketone, and 7-hydroxy-1(3H)-isobenzofuranone as a furanolide.

### 2.2. Cyclobrachycoumarin Inhibited the Proliferation of CRC Cell Lines

**Figure 2:**
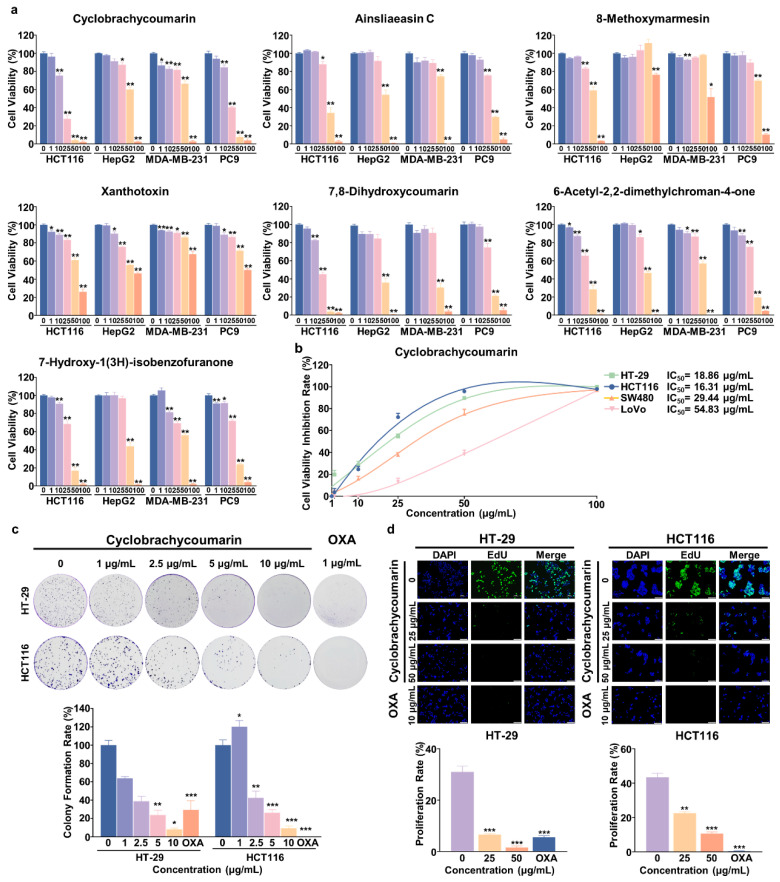
Cyclobrachycoumarin inhibited the proliferation of HT-29 and HCT116 CRC cells. (**a**) Four cancer cell lines (HCT116, HepG2, MDA-MB-231, and PC9) were treated with seven compounds at varying concentrations (0, 1, 10, 25, 50, and 100 μg/mL) for 48 h. (**b**) CRC cell lines HT-29, HCT116, SW480, and LoVo were treated with cyclobrachycoumarin at concentrations varying from 0 to 100 μg/mL for 48 h. (**c**) A colony formation assay valuated the effects of cyclobrachycoumarin (1, 2.5, 5, and 10 μg/mL) and OXA (1 μg/mL) on the clonogenic potential of HT-29 and HCT116 cells. (**d**) Cell proliferation was assessed by an EdU fluorescence imaging assay after treating HT-29 and HCT116 cells with cyclobrachycoumarin (25 and 50 μg/mL) and OXA (10 μg/mL) for 48 h. Proliferating cells are shown in green, while nuclei are shown in blue. Images were taken from randomly selected fields for analysis. Scale bars represent 50 μm. Histograms display mean cell proliferation rates. * *p* < 0.05, ** *p* < 0.01, and *** *p* < 0.001. Each experiment was performed in triplicate. OXA: oxaliplatin, a first-line chemotherapeutic agent, which serves as a positive control.

Previous studies have reported anti-tumor activity in *G. piloselloides* extracts [27]. First, the seven isolated compounds were evaluated for their anti-tumor potential against four common cancer cell lines: HCT116 (CRC), HepG2 (HCC), MDA-MB-231 (BC), and PC9 (LC). Most compounds showed moderate anti-tumor effects at concentrations of 25–50 μg/mL, while xanthotoxin exhibited activity at higher concentrations (50–100 μg/mL). Notably, cyclobrachycoumarin demonstrated significant anti-tumor activity against HCT116 cells, with an IC_50_ value of 16.31 μg/mL (Figure 2a and Table 1). Given its notable anti-CRC effects, cyclobrachycoumarin was selected for further analysis.

To further explore its anti-CRC potential, cyclobrachycoumarin’s impact on cell viability was tested across several CRC cell lines (HT-29, HCT116, SW480, and LoVo) and compared to OXA, a standard clinical anti-tumor agent. The results showed a dose-dependent inhibition of cell proliferation, with varying sensitivities among the CRC cell lines. Cyclobrachycoumarin was most effective in HT-29 and HCT116 cells, achieving IC_50_ values of 18.86 and 16.31 μg/mL, respectively. In contrast, SW480 and LoVo cells showed higher IC_50_ values (29.44 and 54.83 μg/mL, respectively), indicating less sensitivity. Notably, in HT-29 cells, cyclobrachycoumarin’s IC_50_ value was lower than that of OXA (Figure 2b and Table 2). Thus, HT-29 and HCT116 cells were selected for further investigation. Consistent with these results, both colony formation and EdU incorporation assays confirmed that cyclobrachycoumarin suppressed proliferation in HT-29 and HCT116 cells in a dose-dependent manner (Figure 2c,d). These findings indicate that cyclobrachycoumarin exerts significant anti-proliferative effects on CRC cells in vitro.

### 2.3. Cyclobrachycoumarin Induced Cell Cycle Arrest and Apoptosis in CRC Cell Lines

**Figure 3:**
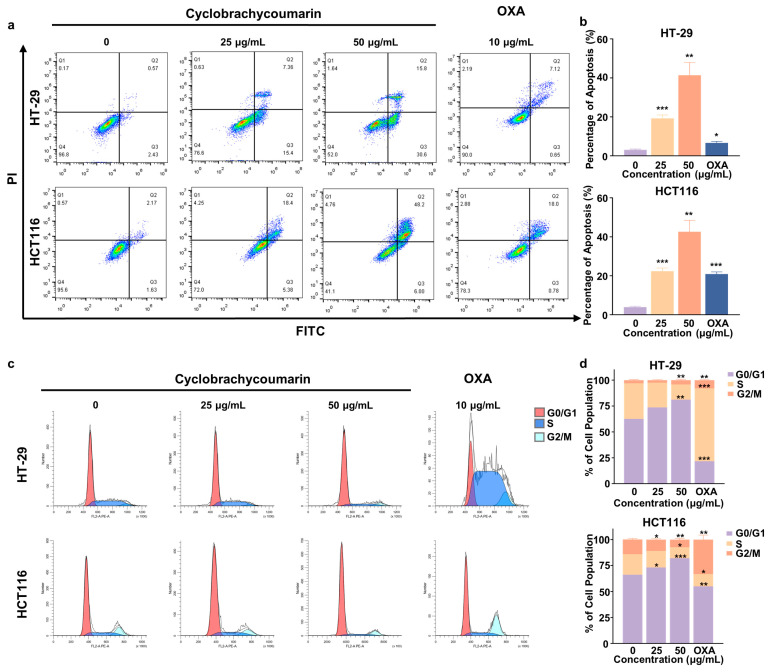
Cyclobrachycoumarin inhibited cell cycle progression and induced apoptosis in CRC cells. (**a**,**b**) HT-29 and HCT116 cells were treated with cyclobrachycoumarin (25 and 50 μg/mL) and OXA (10 μg/mL) for 48 h, and apoptosis was analyzed using flow cytometry with Annexin V-FITC/PI dual staining. (**c**,**d**) Cell cycle progression in HT-29 and HCT116 cells was assessed by flow cytometry after treatment with cyclobrachycoumarin (25 and 50 μg/mL) and OXA (10 μg/mL) for 48 h. * *p* < 0.05, ** *p* < 0.01, and *** *p* < 0.001. Each experiment was performed in triplicate.

Tumor cell progression is closely linked to dysregulated apoptosis and cell cycles control, which support tumor cell proliferation and are key targets for anti-tumor therapies [28,29]. In this study, apoptosis and cell cycle distribution were assessed by flow cytometry. Treatment with cyclobrachycoumarin significantly increased apoptosis in both HT-29 and HCT116 cells. At a concentration of 50 μg/mL, cyclobrachycoumarin induced apoptosis in 41.42% of HT-29 cells and 42.62% of HCT116 cells (Figure 3a,b). Cyclobrachycoumarin also induced a concentration-dependent G1 phase arrest in both cells. In HT-29 cells, treatment with 50 μg/mL cyclobrachycoumarin increased the percentage of cells in the G1 phase from 62.57% to 81.12%, accompanied by a reduction in cells in the S and G2/M phases. Similar results were observed in HCT116 cells, with a significant increase in the G1 phase population (Figure 3c,d). These findings indicate that cyclobrachycoumarin inhibits CRC cell proliferation by inducing apoptosis and cell cycle arrest.

### 2.4. Cyclobrachycoumarin Inhibited CRC Cell Proliferation Through the Generation of ROS and the Modulation of Apoptosis and Cell Cycle-Related Pathways

**Figure 4:**
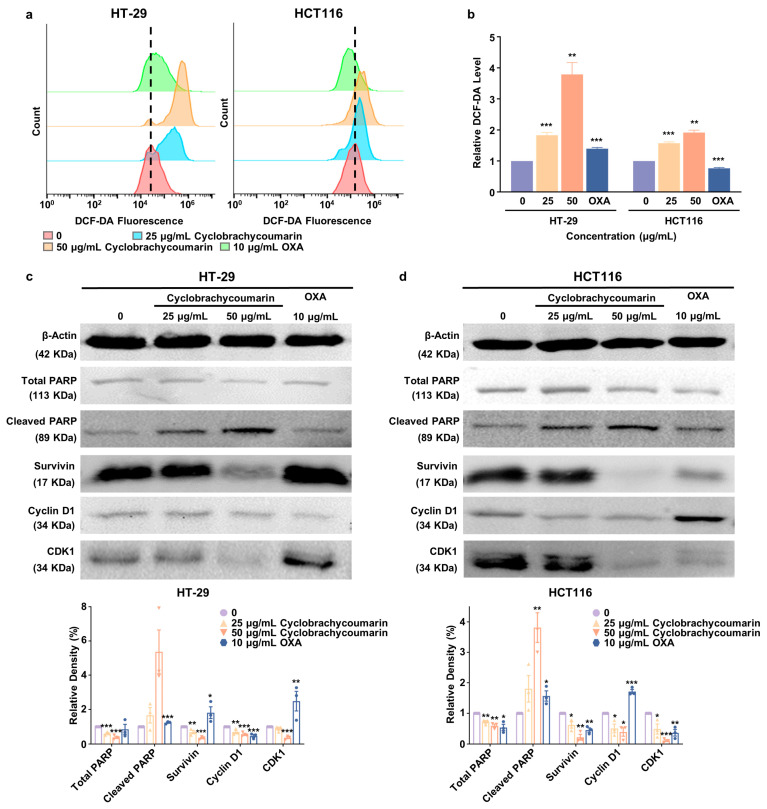
Mechanism of cyclobrachycoumarin-induced cell cycle arrest and apoptosis in CRC cells. (**a**,**b**) ROS levels in HT-29 and HCT116 cells were measured by flow cytometry following treatment with cyclobrachycoumarin (25 and 50 μg/mL) and OXA (10 μg/mL) for 48 h. (**c**,**d**) Western blot analysis was performed to assess protein expression levels of total PARP, cleaved PARP, survivin, cyclin D1, and CDK1, which were quantified in HT-29 and HCT116 cells following the incubation of cyclobrachycoumarin (25 and 50 μg/mL) and OXA (10 μg/mL). * *p* < 0.05, ** *p* < 0.01, and *** *p* < 0.001. Each experiment was performed in triplicate.

Evidence indicates that ROS and their derivatives can induce apoptosis [30]. To investigate whether cyclobrachycoumarin-induced apoptosis involves ROS generation, the intracellular ROS level was measured. After 48 h of cyclobrachycoumarin treatment, a substantial increase in ROS levels was observed in both HT-29 and HCT116 cells. Specifically, the ROS level rose 3.8-fold (*p* < 0.01) in HT-29 cells and 1.9-fold (*p* < 0.01) in HCT116 cells at a cyclobrachycoumarin concentration of 50 μg/mL (Figure 4a,b). Cyclobrachycoumarin treatment also affected the expressions of key apoptosis markers, including cleaved PARP and survivin. As shown in Figure 4c,d, the level of cleaved PARP in HT-29 cells increased 1.7-fold at 25 μg/mL and 5.1-fold at 50 μg/mL, while HCT116 cells showed increases of 1.8-fold and 3.8-fold, respectively. In contrast, survivin expression significantly decreased in both cell lines (Figure 4c,d). Together, these results suggest that cyclobrachycoumarin induces apoptosis via ROS accumulation and regulation of cleaved PARP and survivin expressions.

To elucidate the molecular mechanism by which cyclobrachycoumarin induces cell cycle arrest, we analyzed the expression levels of key cell cycle-related proteins, including cyclin D1 and CDK1 in CRC cells, following cyclobrachycoumarin treatment [31]. Results indicated that cyclobrachycoumarin reduced cyclin D1 and CDK1 expressions in a dose-dependent manner after 48 h of treatment at concentrations of 25 and 50 μg/mL. At a concentration of 50 μg/mL, cyclin D1 and CDK1 levels decreased by 50% and 60% in HT-29 cells, respectively, while reductions of 60% and 90% were observed in HCT116 cells (Figure 4c,d). These findings confirm that cyclobrachycoumarin inhibits CRC cell proliferation by promoting ROS generation, down-regulating the apoptosis inhibitory protein survivin and cell cycle-related proteins cyclin D1 and CDK1, and up-regulating the pro-apoptotic protein cleaved PARP.

### 2.5. Cyclobrachycoumarin Suppressed Tumor Growth in Subcutaneous Xenograft Mice Models

**Figure 5:**
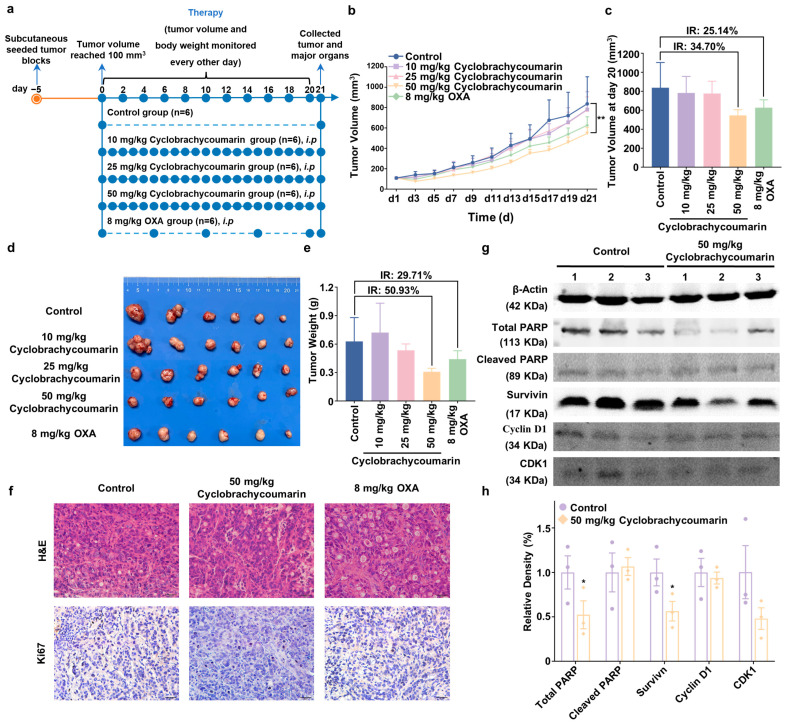
Cyclobrachycoumarin suppressed tumor growth in subcutaneous xenograft mice models. (**a**) Schematic illustration of the pharmacodynamic evaluation of cyclobrachycoumarin in an HT-29 CRC xenograft model. (**b**,**c**) Tumor volume progression over the course of the experiments and the inhibition rate (IR) at the end of the experiment. (**d**,**e**) Tumor weight in mice treated with different concentrations of cyclobrachycoumarin or OXA, along with the IR at the end of the experiment. (**f**) H&E staining and immunohistochemical analysis of Ki67 expression were performed on tumor tissues following treatment with cyclobrachycoumarin (50 mg/kg) or OXA (8 mg/kg). (**g**,**h**) Three tumor samples were randomly selected from each group (control and 50 mg/kg cyclobrachycoumarin) for Western blot analysis. The expression levels of total PARP, cleaved PARP, survivin, cyclin D1, and CDK1 were assessed. * *p* < 0.05 and ** *p* < 0.01.

The above results confirmed cyclobrachycoumarin’s anti-CRC effect in vitro, yet in vivo validation was required. Therefore, CRC xenograft models were established in nude mice by inoculating them with HT-29 cells. Once tumors reached a volume of 100 mm^3^, the mice were randomized into five groups for a 20-day treatment period. Cyclobrachycoumarin (10, 25, and 50 mg/kg) was administered intraperitoneally once daily, while OXA (8 mg/kg) was used as a positive control and administered intraperitoneally every five days (Figure 5a). Consistent with in vitro findings, cyclobrachycoumarin effectively inhibited CRC progression in vivo. In the 50 mg/kg cyclobrachycoumarin group, tumor volume inhibition reached 34.70%, while tumor weight inhibition was 50.93%, compared to the inhibition rates of 25.14% and 29.71%, respectively, in the OXA group (Figure 5b–e). H&E and IHC analyses further confirmed cyclobrachycoumarin’s anti-CRC effect (Figure 5f). Additionally, Western blot analysis of tumor tissue indicated that 50 mg/kg cyclobrachycoumarin decreased the expressions of CDK1, total PARP, and survivin while enhancing cleaved PARP levels in vivo (Figure 5g,h). These results suggest that cyclobrachycoumarin suppresses CRC tumor growth in vivo by modulating cell cycle progression and promoting apoptosis.

**Figure 6:**
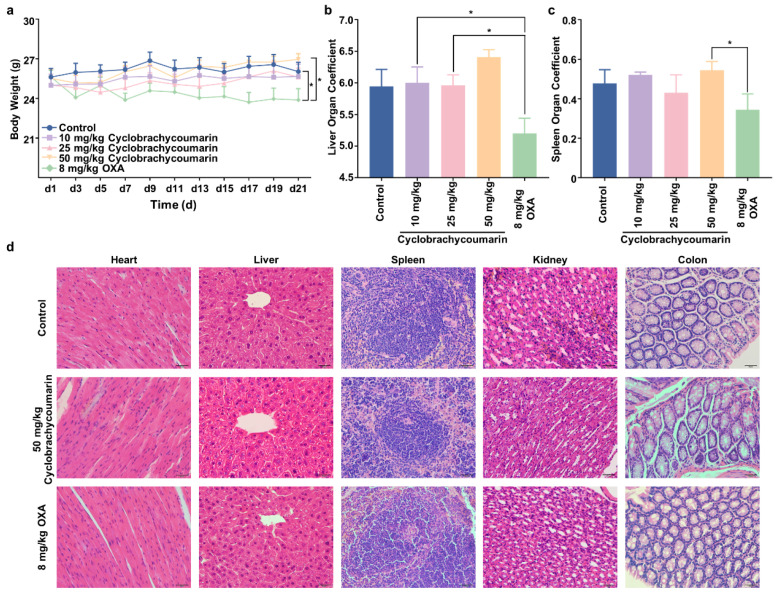
In vivo biological safety evaluation of cyclobrachycoumarin. (**a**) Body weight of mice monitored through the experiment. (**b**,**c**) Livers and spleens were collected, weighed, and analyzed for organ coefficients per group. (**d**) H&E staining was conducted on heart, liver, spleen, kidney, and colon tissues of mice treated with cyclobrachycoumarin (50 mg/kg) or OXA (8 mg/kg). * *p* < 0.05.

Moreover, while OXA treatment significantly reduced body weight in mice, cyclobrachycoumarin had no such effect (Figure 6a). The liver and spleen organ coefficients also remained unchanged in the cyclobrachycoumarin treatment group (Figure 6b,c). Histological analysis showed that cyclobrachycoumarin did not disrupt the structural integrity of the heart, liver, spleen, kidney, or colon tissues (Figure 6d). Collectively, these findings suggest that cyclobrachycoumarin inhibits CRC tumor growth with favorable tolerability in vivo.

## 3. Discussion

CRC is a prevalent form of cancer globally [3]. Although chemotherapy remains the preferred treatment for advanced CRC, its high toxicity and limited effectiveness often fall short of clinical needs [32]. Therefore, the development of safer and more efficient therapeutic options is essential. In this study, we extracted seven compounds from *G. piloselloides* and evaluated their anti-tumor activity. Among these, cyclobrachycoumarin demonstrated significant inhibitory effects on CRC cells, inducing apoptosis and cell cycle arrest. This activity was mediated through the generation of ROS and down-regulations of the anti-apoptotic protein survivin and cell cycle regulators cyclin D1 and CDK1, along with the up-regulation of the pro-apoptotic protein cleaved PARP. In summary, cyclobrachycoumarin shows promise as a potential therapeutic agent for CRC treatment.

*G. piloselloides* is a common herb in southwestern China, traditionally used to treat fever and cough [33]. Previous studies have shown that coumarins are the primary constituents of *G. piloselloides*, with potential anti-tumor activity [18,34]. However, few pharmacological studies have explored the specific bioactive components of *G. piloselloides* and their anti-tumor effects. In this study, we isolated seven compounds from *G. piloselloides*, including five coumarins (Figure 1). Notably, cyclobrachycoumarin exhibited significant anti-tumor effects on CRC cells, demonstrating superior efficacy (Figure 2 and Table 1). Although cyclobrachycoumarin was previously isolated from *Subtribe Mutisiinae* by *H. Robinson* et al. and from *G. piloselloides* by Zhongmei Zou et al., its anti-tumor properties have not yet been investigated [20,21]. Consequently, we selected cyclobrachycoumarin for the evaluation of its anti-CRC potential and mechanistic action.

In this study, the five coumarins showed strong inhibitory effects on HCT116 cells, with cyclobrachycoumarin and 7,8-dihydroxycoumarin demonstrating greater potency than ainsliaeasin C, 8-methoxymarmesin, and xanthotoxin. These differences in efficacy may be attributed to variations in the position and number of hydroxyl, methoxy, and other functional groups [35], which are known to influence their biological activities [36]. Furthermore, the anti-tumor effects of the five coumarins in this study differed from those reported by Zou et al. [20], who investigated gerberchromone D and gerbeloid J. Their findings, based on preliminarily CCK-8 assay results, provided only limited insights into the anti-tumor properties of these compounds. In contrast, our results provided stronger evidence that cyclobrachycoumarin effectively inhibited CRC development both in vitro and in vivo, with favorable tolerability. This positions cyclobrachycoumarin as a promising candidate for clinical CRC therapy.

Aberrant cell cycle progression and apoptosis resistance are hallmark features of tumor cells and are widely recognized as key anti-tumor targets [37]. In this study, we observed that cyclobrachycoumarin induced cell cycle arrest and apoptosis in CRC cells in vitro (Figure 3). Reported markers of apoptosis included intracellular ROS level, survivin, and cleaved PARP [38,39,40], while cell cycle progression was regulated by specific cyclin-CDK complexes, such as cyclin D1 and CDK4/6 for the G1 phase, cyclin A2 and CDK2 for the S phase, and cyclin B1 and CDK1 for the G2/M phase [41]. To further explore the underlying mechanism of cyclobrachycoumarin’s effects, we examined changes in cell apoptosis and cell cycle-related markers. We found that cyclobrachycoumarin may induce cell cycle arrest in CRC cells by downregulating cyclin D1 and CDK1 expressions. Additionally, it promotes apoptosis through ROS accumulation, up-regulation of cleaved PARP, and down-regulation of survivin (Figure 4), thereby suggesting a potential mechanism for its anti-CRC activity.

Given the strong anti-tumor activity of cyclobrachycoumarin observed in vitro, we further investigated its efficacy in vivo using xenograft transplantation models. Remarkably, treatment with 50 mg/kg cyclobrachycoumarin demonstrated superior tumor inhibition, compared to the positive control, OXA. Consistent with our in vitro findings, cyclobrachycoumarin at this dosage reduced the expressions of survivin and CDK1 while increasing cleaved PARP expression (Figure 5). Additionally, while OXA treatment led to a reduction in body weight and liver organ coefficient in mice, cyclobrachycoumarin administration did not produce these adverse effects, suggesting that cyclobrachycoumarin may be an effective and safe candidate for anti-CRC therapy.

In this study, we elucidated the anti-CRC activity and mechanism of cyclobrachycoumarin, a compound isolated from *G. piloselloides*. However, one limitation of our findings is that the subcutaneous transplantation tumor model does not fully mimic the progression of CRC. To address this, future studies will utilize a patient-derived orthotopic transplantation tumor model to more accurately evaluate cyclobrachycoumarin’s anti-CRC efficacy. Additionally, while our results showed that cyclobrachycoumarin was well tolerated in mice, further rigorous studies on its biosafety are essential to support its potential for clinical application. In conclusion, our findings suggest that cyclobrachycoumarin inhibits CRC progression through mechanisms involving cell cycle arrest and apoptosis, with a favorable tolerability profile, positioning it as a promising candidate for safe and effective CRC therapy.

## 4. Materials and Methods

### 4.1. Plant Material

The *G. piloselloides* was purchased from Hebei Kang Yuan Ben Cao Trading Company (Baoding, Hebei, China) in November 2022. The sample identity was verified by Professor Yuan-Huo Dong (College of Life Science, Jianghan University). A reference specimen with the designated GP-20220519 was stored at the Institute of Biomedical Sciences, School of Medicine, Jianghan University.

### 4.2. Extraction and Isolation

*G. piloselloides* (20 kg) was subjected to three extractions with MeOH (methanol) at a three-day interval. The solvent was enriched under vacuum to obtain a crude extract of 1.0 kg. Next, the crude extract was separated by silica gel chromatography using eluents of CH_2_Cl_2_ (dichloromethane), EtOAc (ethyl acetate), and MeOH to yield 2 different fractions (Fr.1 and Fr.2). Fr.1 (150 g) was then chromatographed on a silica gel and washed with PE (petroleum ether)/EtOAc (100:0, 9:1, 8:2, 60:40, 7:3, 6:4, 1:1, and 0:100) to yield 8 fractions (Fr.1.1−Fr.1.8). Fr.1.1 (32.5 g) was then separated on an ODS column and eluted with the mixture of MeOH/H_2_O (70:30, 80:20, 90:10, and 100:0) to yield 4 fractions (Fr.1.1.1–Fr.1.1.4). Cyclobrachycoumarin (646.5 mg), ainsliaeasin C (12.5 mg), xanthotoxin (10.0 mg), 6-acetyl-2,2-dimethylchroman-4-one (5.7 mg), and 7-hydroxy-1(3H)-isobenzofuranone (3.6 mg) were isolated from Fr.1.1.3 (1.25 g) by semi-preparative HPLC (CAN−H_2_O, 80:20, 4.0 mL/min). Fr.2 (62.5 g) was separated by an ODS column and eluted with MeOH/H_2_O (55:45, 65:35, 75:25, 85:15, 95:5, and 100:0) to yield 6 fractions (Fr.2.1–Fr.2.6). Fr.2.2 (13.6 g) was separated on a Sephadex LH-20 column, and the MeOH was used for the eluting mobile phase to yield 6 fractions (Fr.2.2.1–Fr.2.2.6). The 7,8-dihydroxycoumarin (5.3 mg) was purified from Fr.2.2.2 (124.6 mg) by semi-preparative HPLC (MeOH−H_2_O, 45:55, 3.0 mL/min), and 8-methoxymarmesin (6.5 mg) was purified from Fr.2.2.3 (68.6 mg) by semi-preparative HPLC (CAN−H_2_O, 53:47, 3.0 mL/min).

### 4.3. Cell Lines and Cell Culture

MDA-MB-231 (BC), HepG2 (HCC), PC9 (LC), HCT116 (CRC), HT-29 (CRC), SW480 (CRC), and LoVo (CRC) cells were obtained from the ATCC (Manassas, VA, USA). These cells were cultured in DMEM (Gibco, Jenks, OK, USA) with 10% FBS (Gibco, Jenks, OK, USA) and 1% penicillin/streptomycin (Beytime, Shanghai, China) at 37 °C in an incubator with 5% CO_2_.

### 4.4. Cell Viability Assay

The cells were incubated with the *G. piloselloides* extracts and OXA (a first-line chemotherapeutic agent; Selleck, Houston, TX, USA). Cell viability was assessed at 48 h using a CCK-8 kit (Biosharp, Hefei, China). Cell viability was computed by the formula: Cell viability (%) = $\frac{OD\;(Treatment)-OD\;(Blank)}{OD\;\left(Control\right)-OD\;(Blank)}$ × 100%. The OD (Treatment) and OD (Control) denote the absorbance of extracts/OXA-treated cells and culture medium-treated cells, respectively; the OD (Blank) denotes the absorbance of blank cells.

### 4.5. Colony Formation Assay

CRC cells were inoculated with cyclobrachycoumarin and OXA over a period of 14 days. The remaining colonies were visualized with crystal violet and enumerated by Image J V1.54 software (Perkin Elmer, Shelton, CT, USA).

### 4.6. EdU Incorporation Assay

Following the treatment of CRC cells with cyclobrachycoumarin and OXA, an EdU imaging detection kit (KevGEN BioTECH, Nanjing, China) was utilized. Initially, cells were cultured with 10 μM 5-ethynyl-2′-deoxyuridine (EdU) and immobilized with paraformaldehyde. Then, the cells were subjected to Triton X-100 permeabilization and incubated with a 1 × Click-iT reactant mixture in darkness. Lastly, the cells were embedded in antifade mounting medium with DAPI (Beytime, Shanghai, China) and photographed in multiple fields by an inverted fluorescence microscope (Motic, Xiamen, China). The number of proliferating cells (green) and nuclei (blue) were counted by Image J V1.54j software (Perkin Elmer, Shelton, CT, USA).

### 4.7. Cell Apoptosis Analysis

CRC cells were treated with cyclobrachycoumarin and OXA for 48 h. Then, cells were harvested and stained following the steps in the Annexin V-FITC/PI apoptosis kit (MULTI SCIENCES, Hangzhou, China). Apoptosis was measured by a flow cytometer (Accuri™ C6, BD Biosciences, San Jose, CA, USA). Data were analyzed using FlowJo V10 software (BD Biosciences, San Jose, CA, USA).

### 4.8. Cell Cycle Analysis

CRC cells were treated with cyclobrachycoumarin and OXA. The cells were collected and stained by a cell cycle staining kit (MULTI SCIENCES, Hangzhou, China) under dark conditions. The cells were analyzed by flow cytometry (Accuri™ C6, San Jose, CA, BD Biosciences), and data were characterized using Modfit LT 5.0 software (Verity Software House, Topsham, ME, USA).

### 4.9. ROS Accumulation Assay

The ROS was assessed by 2′,7′-dichlorofluorescein diacetate (DCFH-DA) staining. Cells were collected after 48 h of treatment with cyclobrachycoumarin or OXA. The cells were stained with the ROS assay kit (Beytime, Shanghai, China) and analyzed by flow cytometry (Accuri™ C6, San Jose, CA, BD Biosciences). Data analysis was performed with FlowJo V10 software (BD Biosciences, San Jose, CA, USA).

### 4.10. Western Blot Analysis

After a 48 h treatment with cyclobrachycoumarin and OXA, the cells were solubilized with lysis buffer (Beytime, Shanghai, China), which contained a comprehensive mixture of protease and phosphatase inhibitors (Beytime, Shanghai, China). A total of 25 µg of lysates was loaded onto a 12% SDS-PAGE gel and blotted onto an NC membrane (Pall, Port Washington, NY, USA). Membranes were incubated with specific antibodies, including anti-PARP antibody (Proteintech, Wuhan, China), anti-cleaved PARP antibody (Cell Signaling, Danvers, MA, USA), anti-survivin antibody (Cell Signaling, Danvers, MA, USA), anti-cyclin D1 antibody (Proteintech, Wuhan, China), anti-CDK1 antibody (Chengdu Zen Biotechnology, Chengdu, China), and anti-actin antibody (HUABIO, Hangzhou, China). The membranes were immersed in IRDye 800 CW secondary antibody (Licor, Lincoln, NE, USA) and imaged using an Odyssey SA imaging system (Licor, Lincoln, NE, USA). Strips were quantified using Image Studio Ver 5.2 software (Licor, Lincoln, NE, USA).

### 4.11. Xenograft Transplantation Assay

Six-week-old male BALB/c-nu mice were obtained from GemPharmatech Co., Ltd. (Nanjing, China, No. B202311260058). The 5 × 10^6^ HT-29 cells were injected subcutaneously to generate tumors, and the tumors were cut into 1 mm^3^ fragments when they reached 500 mm^3^ in volume. Then, the fragments were implanted subcutaneously, and tumor volume was measured every two days to monitor tumor growth. Once the mean volume of tumors attained 100 mm^3^, the mice were randomized into five groups (6 per group), including the control group, 10 mg/kg cyclobrachycoumarin group, 25 mg/kg cyclobrachycoumarin group, 50 mg/kg cyclobrachycoumarin group, and 8 mg/kg OXA group. Cyclobrachycoumarin was administered intraperitoneally (i.p.) once a day in the cyclobrachycoumarin groups; OXA was administered intraperitoneally (i.p.) every five days. Both tumor volume and body weight measurements were available every two days. Following a 20-day treatment period, mice were euthanized, and the tumors and organs (heart, liver, spleen, kidney, and colon) were preserved in paraformaldehyde and sectioned in paraffin for later study.

The tumor growth inhibition value was calculated based on the following formula: IR (Inhibition Rate) = [1 − $\frac{Tumor\;Volume/Weight\;(Experimental\;Group)}{Tumor\;Volume/Weight\;(Control\;Group)}$] × 100%.

### 4.12. H&E Staining Assay

The above sections were deparaffinized in water and dyed with 10% hematoxylin and 1% eosin. The sections were randomly selected and imaged by an orthoptic microscope (Olympus, Tokyo, Japan).

### 4.13. Immunohistochemistry (IHC) Assay

Immunohistochemistry was performed using the Ki67 antibody (Proteintech, Wuhan, China) on the above paraffin-embedded tumor sections. Sections were incubated with boiling antigen retrieval buffer (Beytime, Shanghai, China) for antigen retrieval. After incubation with the biotinylated secondary antibody (Bosterbio, Wuhan, China), color development was performed using a DAB solution (Beytime, Shanghai, China). Images were captured with an orthogonal microscope (Olympus, Tokyo, Japan).

### 4.14. Statistical Analysis

All experiments were duplicated in triplicate, and data are shown as mean with SEM. Statistics were determined using one-way ANOVA or a two-tailed *t*-test, with *p* < 0.05 defined as being statistically significant. Diagrams were drafted using GraphPad Prism 8.0 (GraphPad, San Diego, CA, USA).

## Figures and Tables

**Table 1:** IC_50_ values (μg/mL) of seven compounds against various cancer cell lines, including HCT116 (CRC), HepG2 (HCC), MDA-MB-231 (BC), and PC9 (LC), over 48 h.

IC_50_ (Mean ± SEM, μg/mL) of Seven Compounds on Different Cancer Cell Lines at 48 h				
Compounds	Colorectal Cancer	Liver Cancer	Breast Cancer	Lung Cancer
	HCT116	HepG2	MDA-MB-231	PC9
Cyclobrachycoumarin	16.31 ± 1.34	53.76 ± 3.46	56.47 ± 5.81	20.82 ± 1.49
Ainsliaeasin C	42.23 ± 2.47	51.35 ± 3.15	57.03 ± 0.84	36.90 ± 1.73
8-Methoxymarmesin	52.28 ± 4.49	>100	>100	60.72 ± 3.50
Xanthotoxin	60.51 ± 4.91	77.83 ± 8.62	>100	>100
7,8-Dihydroxycoumarin	21.44 ± 1.39	41.58 ± 4.13	39.48 ± 3.18	34.48 ± 2.04
6-Acetyl-2,2-dimethylchroman-4-one	32.12 ± 2.59	46.60 ± 2.38	52.22 ± 2.76	33.87 ± 2.60
7-Hydroxy-1(3H)-isobenzofuranone	31.21 ± 1.98	47.61 ± 2.20	42.22 ± 7.02	33.95 ± 1.98

**Table 2:** IC_50_ values (μg/mL) of cyclobrachycoumarin and OXA on four CRC cell lines (HT-29, HCT116, SW480, and LoVo) over 48 h.

Cell Viability IC_50_ (μg/mL) ± SEM				
	Colorectal Cancer			
	HT-29	HCT116	SW480	LoVo
Cyclobrachycoumarin	18.86 ± 3.62	16.31 ± 1.34	29.44 ± 2.56	54.83 ± 4.59
OXA	>25.00	7.58 ± 1.05	12.53 ± 2.1	8.85 ± 1.01

## Data Availability

The data presented in this study are available on request from the corresponding author.

## Supplementary Materials

The following supporting information can be downloaded at: https://www.mdpi.com/article/10.3390/molecules29235678/s1.

## Abbreviations

NMR: nuclear magnetic resonance; HPLC: high-performance liquid chromatography; CRC: colorectal cancer; OXA: oxaliplatin; TCM: traditional Chinese medicine; ROS: reactive oxygen species; FBS: fetal bovine serum; IR: inhibition rate; H&E: hematoxylin–eosin; IHC: immunohistochemistry; CDKs: cyclin-dependent kinases.
