# Supplementary material for: Cyclobrachycoumarin from Gerbera piloselloides Inhibits Colorectal Cancer In Vitro and In Vivo
Source: Molecules. 2024 Nov 30;29(23):5678. doi: 10.3390/molecules29235678 (PMC11643420; doi:10.3390/molecules29235678)
Supplement: Supplementary file 1 [file molecules-29-05678-s001.zip › molecules-3336458-supplementary.pdf]

Supplementary Information for

Cyclobrachycoumarin from *Gerbera piloselloides* Inhibits Colorectal Cancer In Vitro and In Vivo

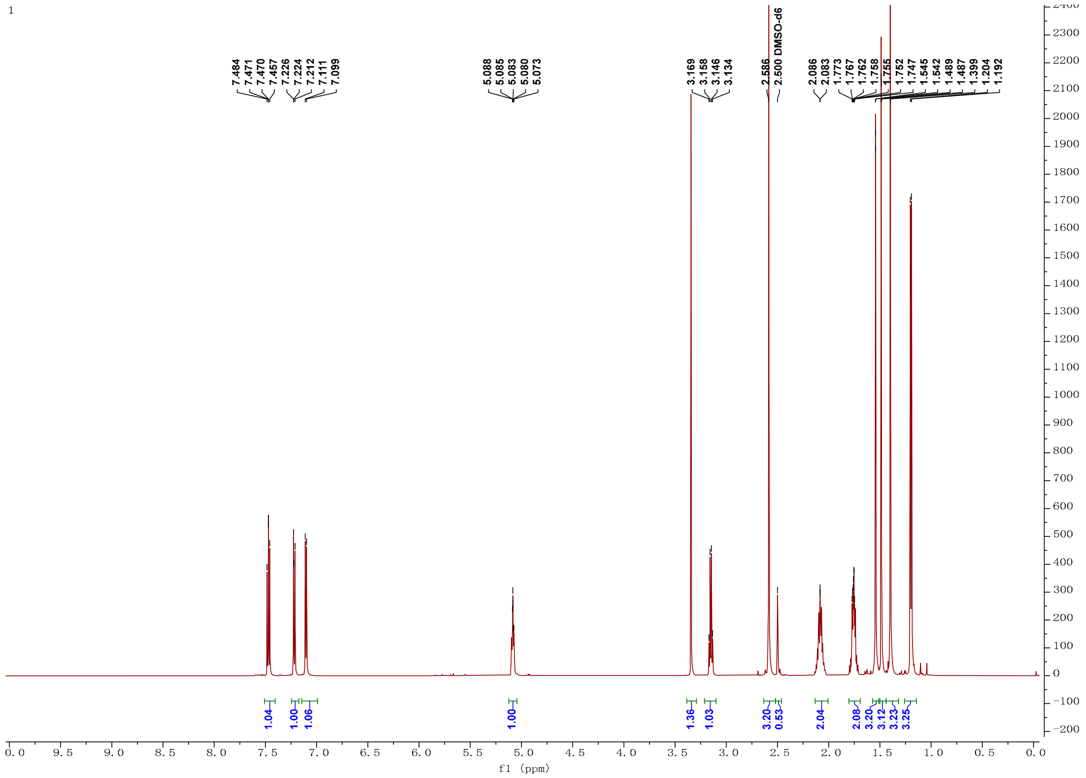

Figure S1. ^{1}H-NMR Spectrum of Cyclobrachycoumarin in DMSO-d_6

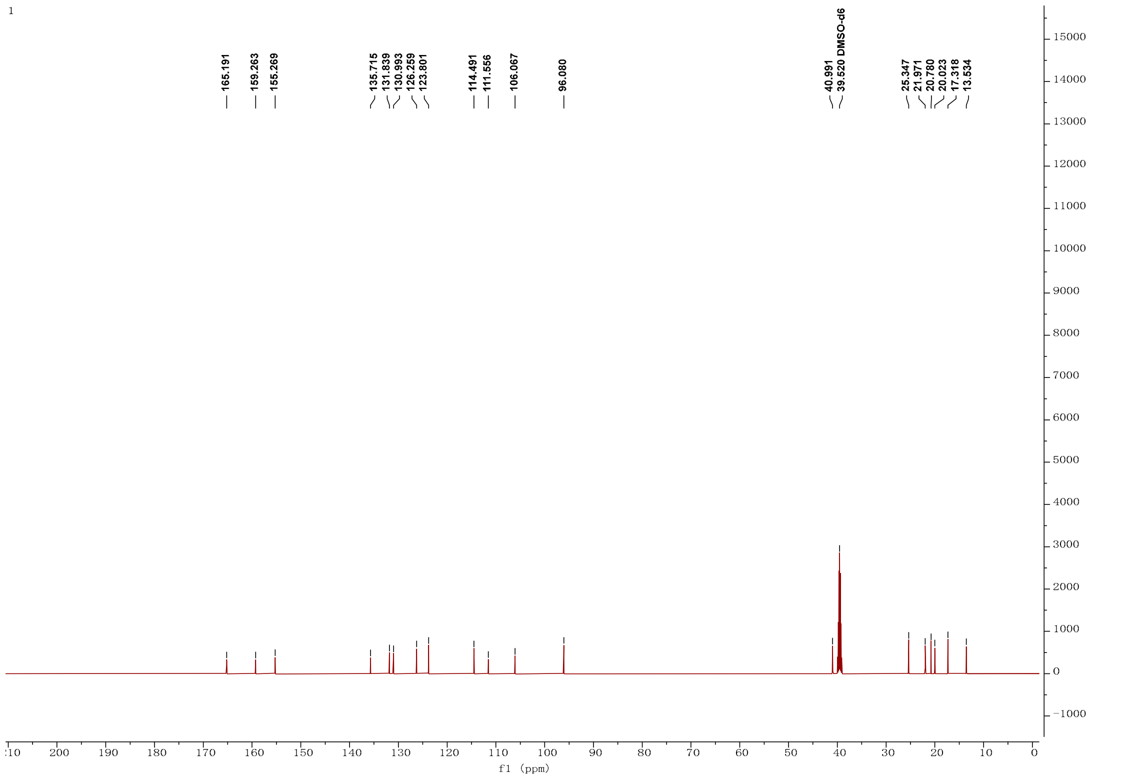

Figure S2. ^{13}C-NMR Spectrum of Cyclobrachycoumarin in DMSO-d_6

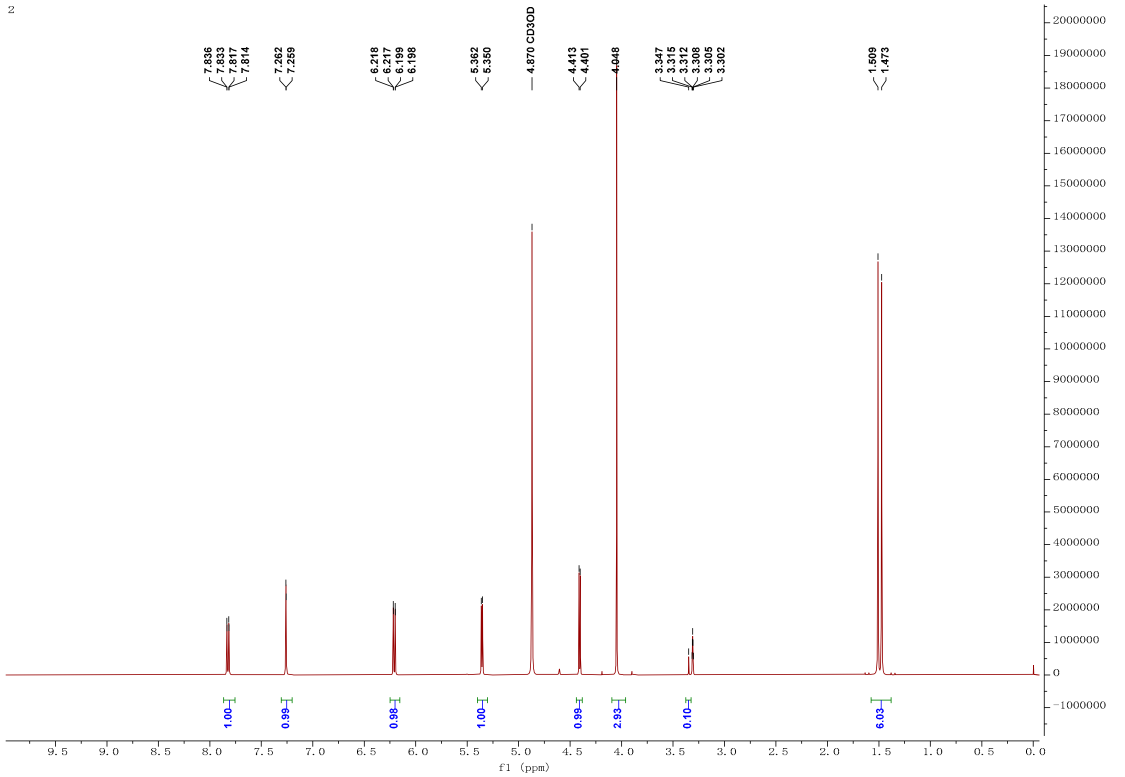

Figure S3. ^{1}H-NMR Spectrum of Ainsliaeasin C in Methanol-d_4

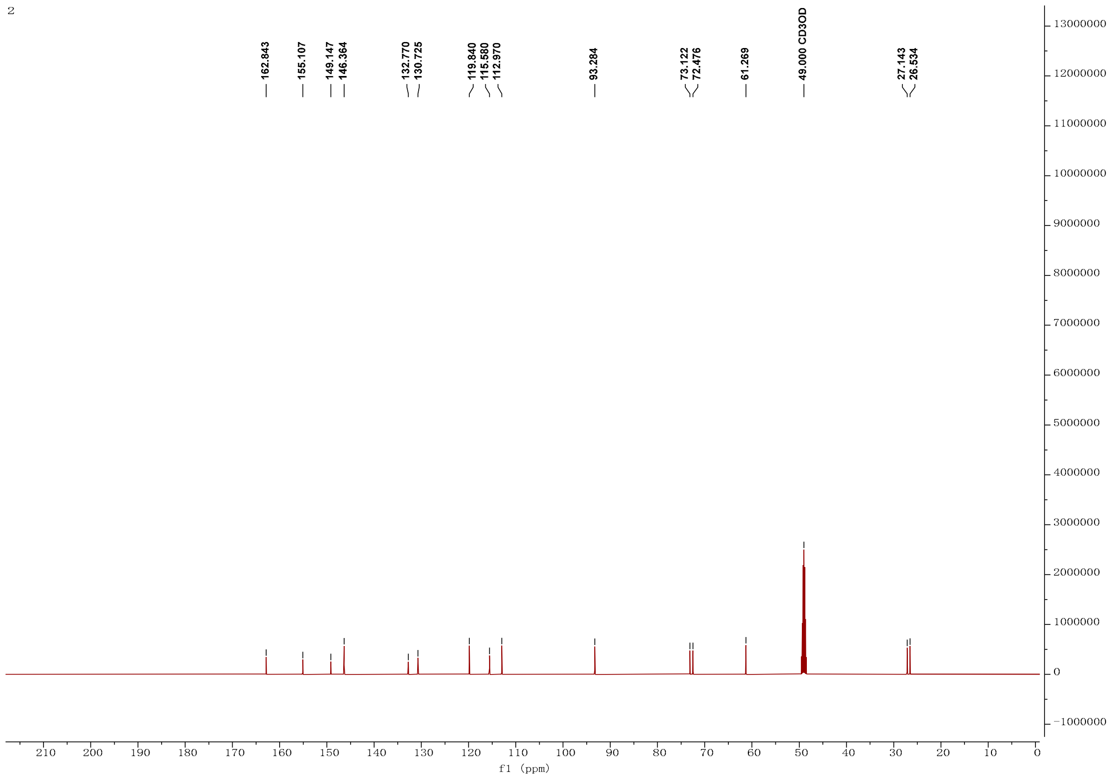

Figure S4. ^{13}C-NMR Spectrum of Ainsliaeasin C in Methanol-d_4

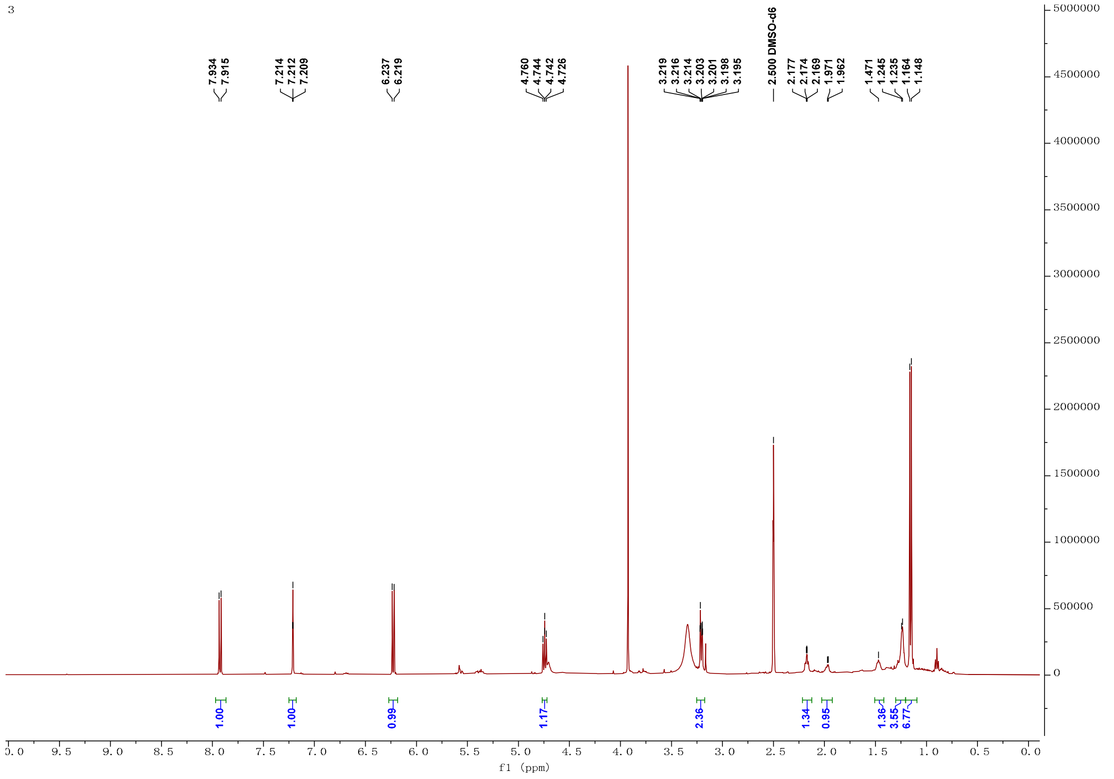

Figure S5. ^{1}H-NMR Spectrum of 8-Methoxymarmesin in DMSO-d_6

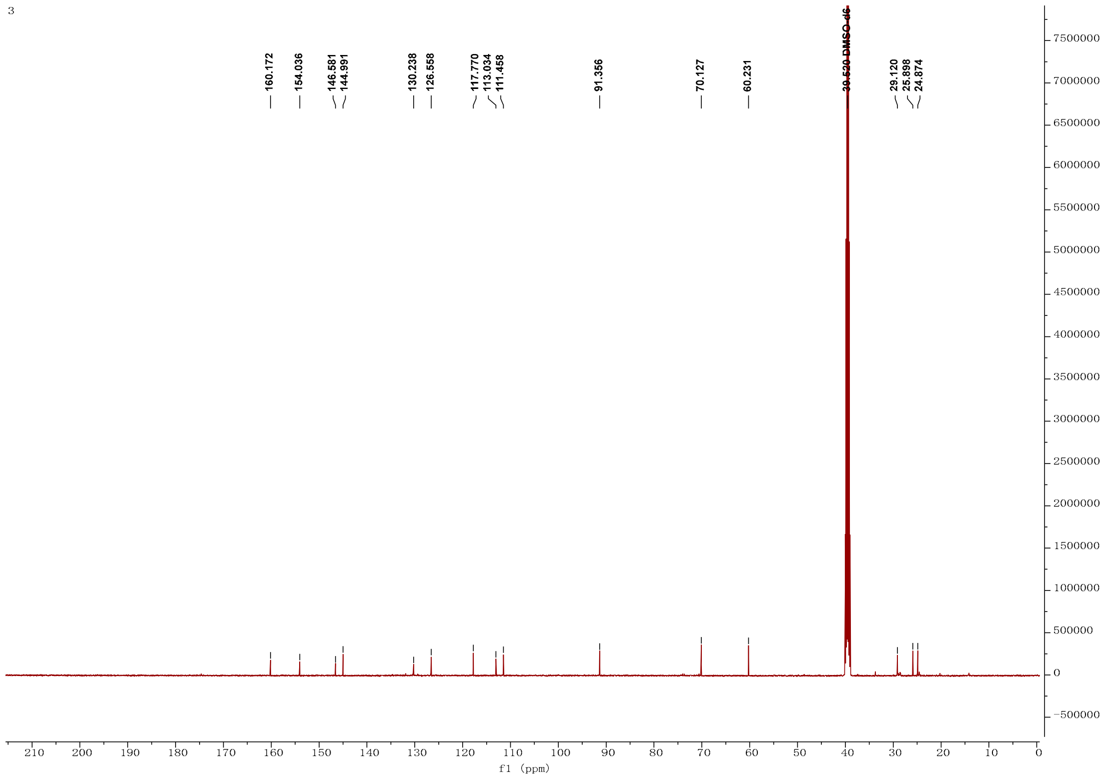

Figure S6. ¹³C-NMR Spectrum of 8-Methoxymarmesin in DMSO-d_6

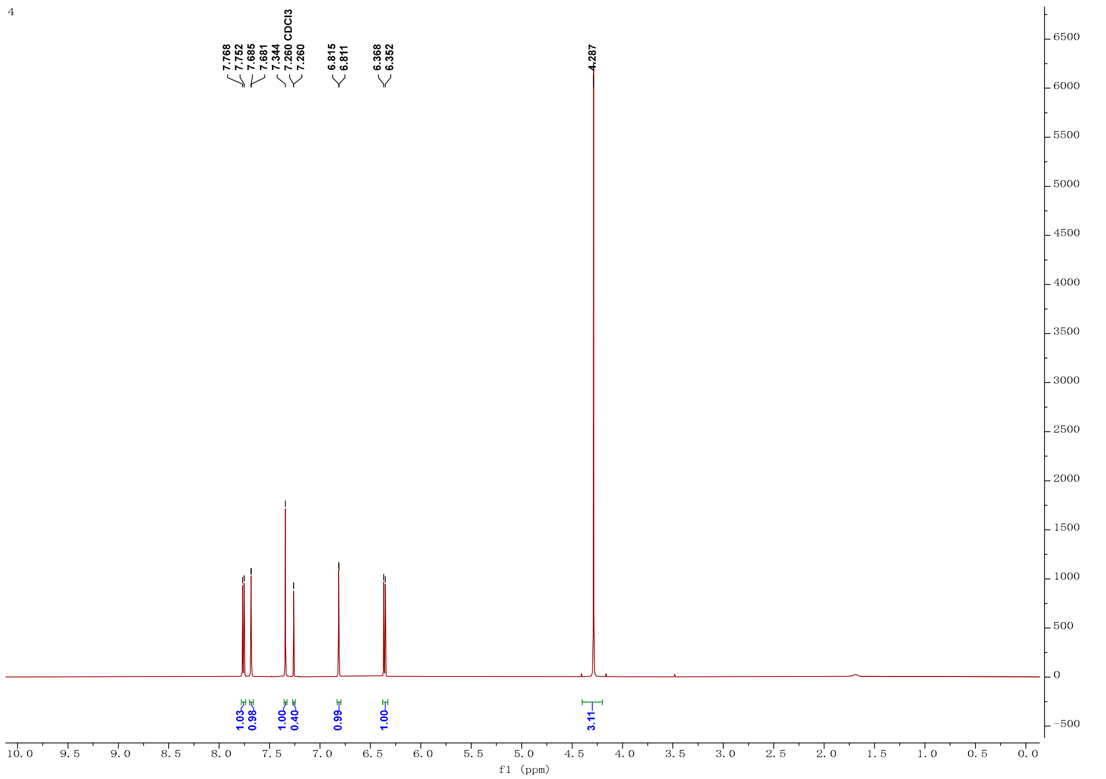

Figure S7. ¹H-NMR Spectrum of Xanthotoxin in $CDCl_3$

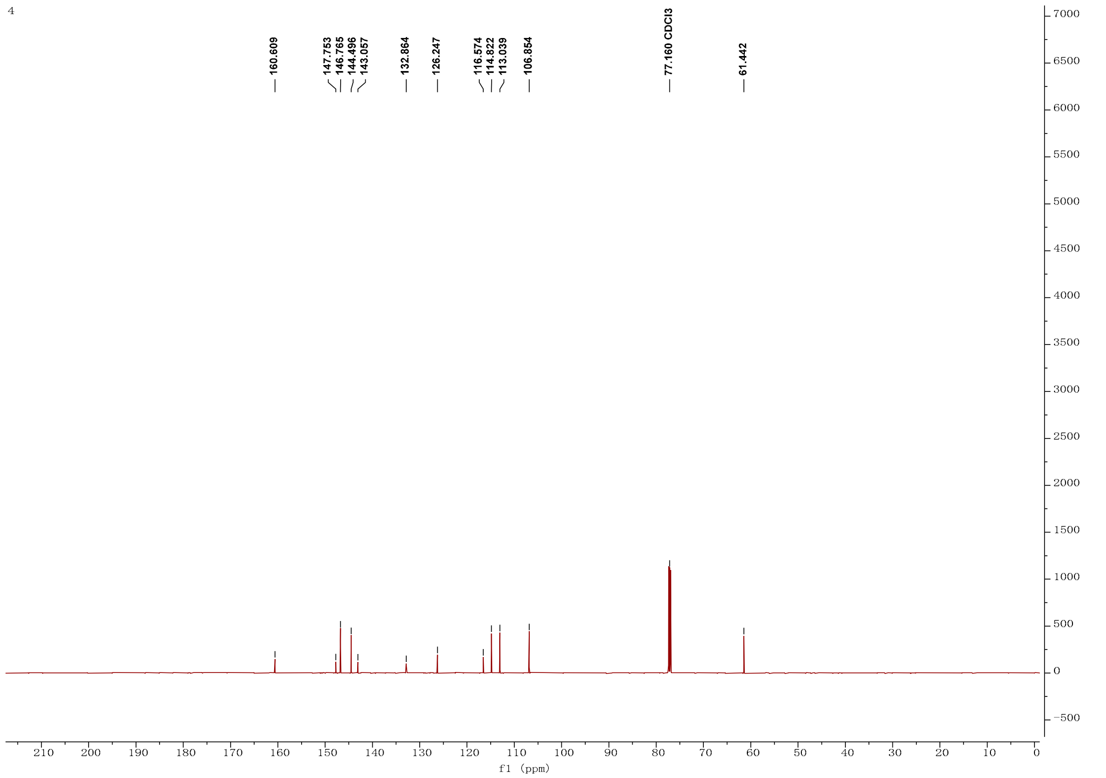

Figure S8. ^{13}C-NMR Spectrum of Xanthotoxin in $CDCl_3$

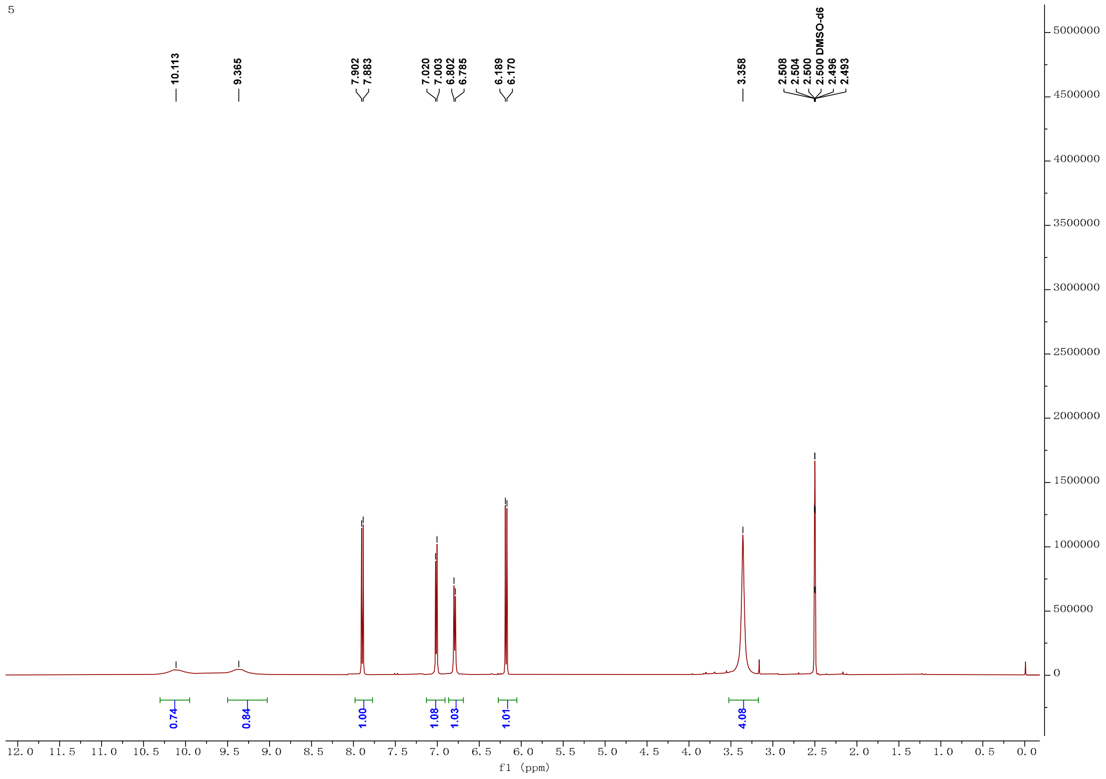

Figure S9. ^{1}H-NMR Spectrum of 7,8-Dihydroxycoumarin in DMSO-d_6

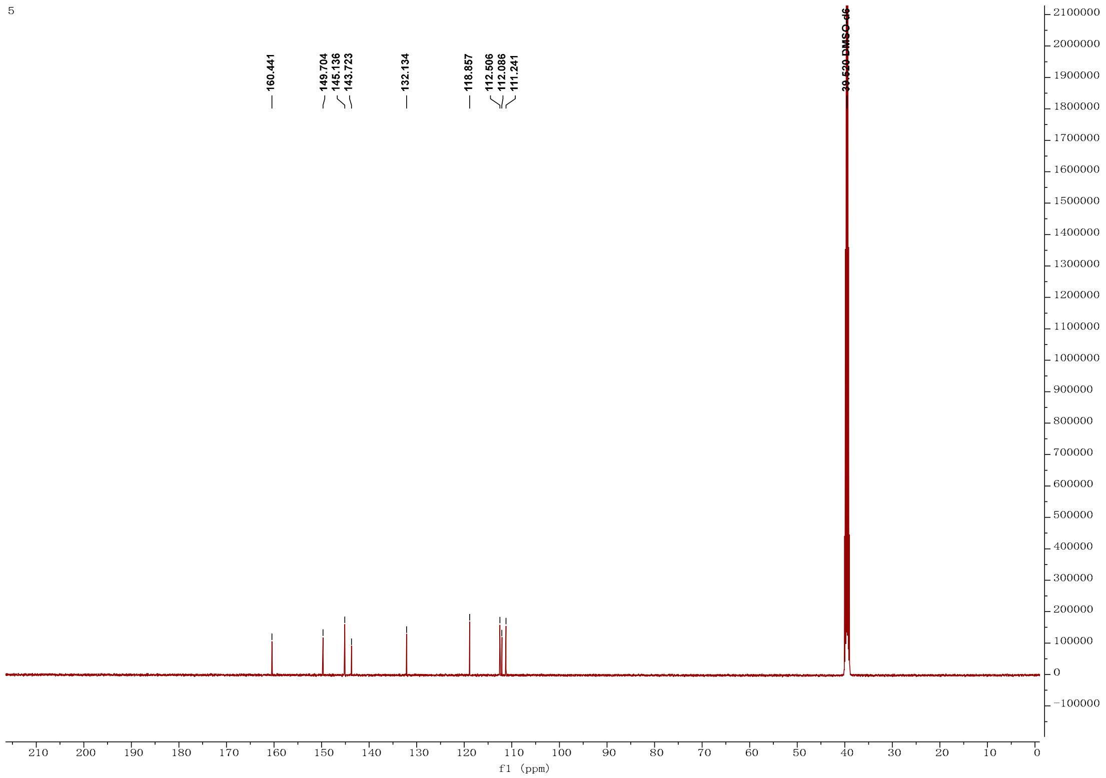

Figure S10. ^{13}C-NMR Spectrum of 7,8-Dihydroxycoumarin in DMSO-d_6

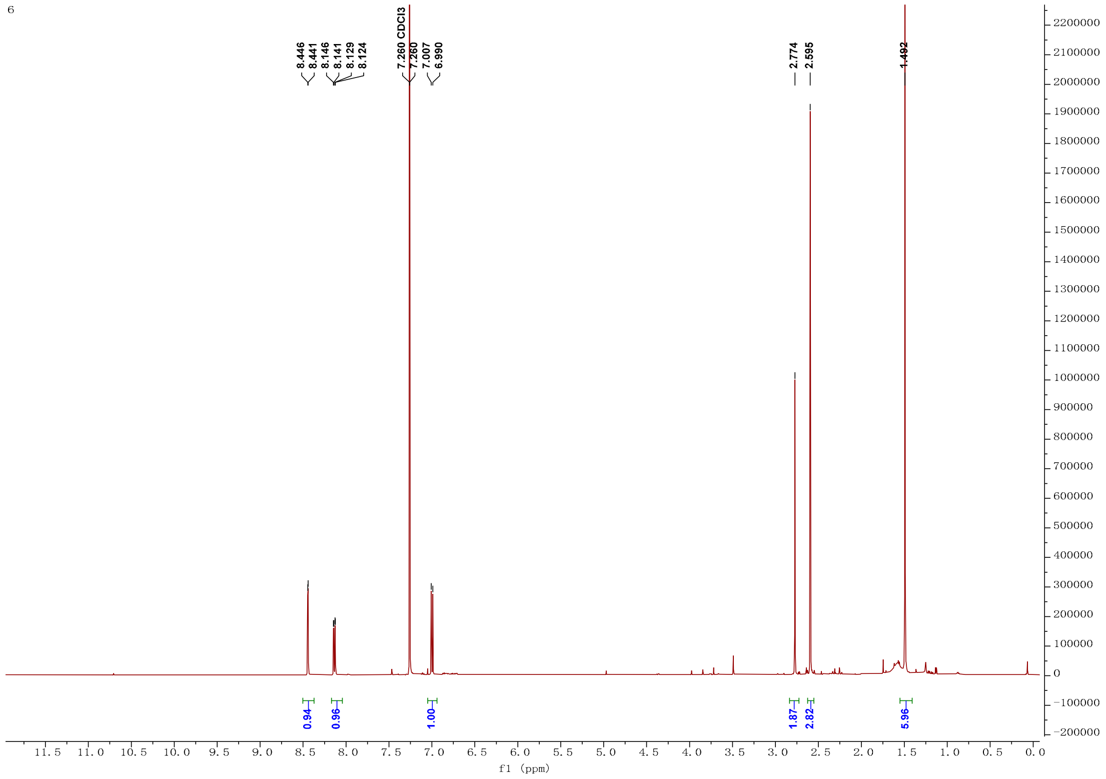

Figure S11. ^{1}H-NMR Spectrum of 6-Acetyl-2,2-dimethylchroman-4-one in $CDCl_3$

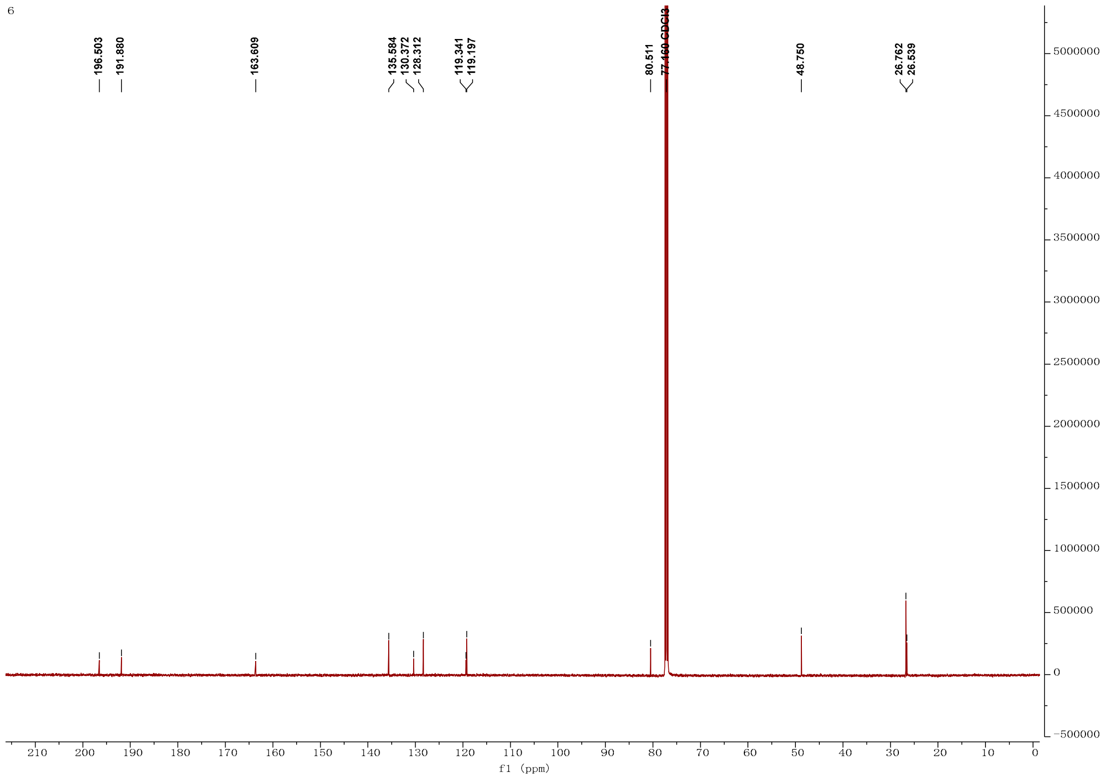

Figure S12. ^{13}C-NMR Spectrum of 6-Acetyl-2,2-dimethylchroman-4-one in $CDCl_3$

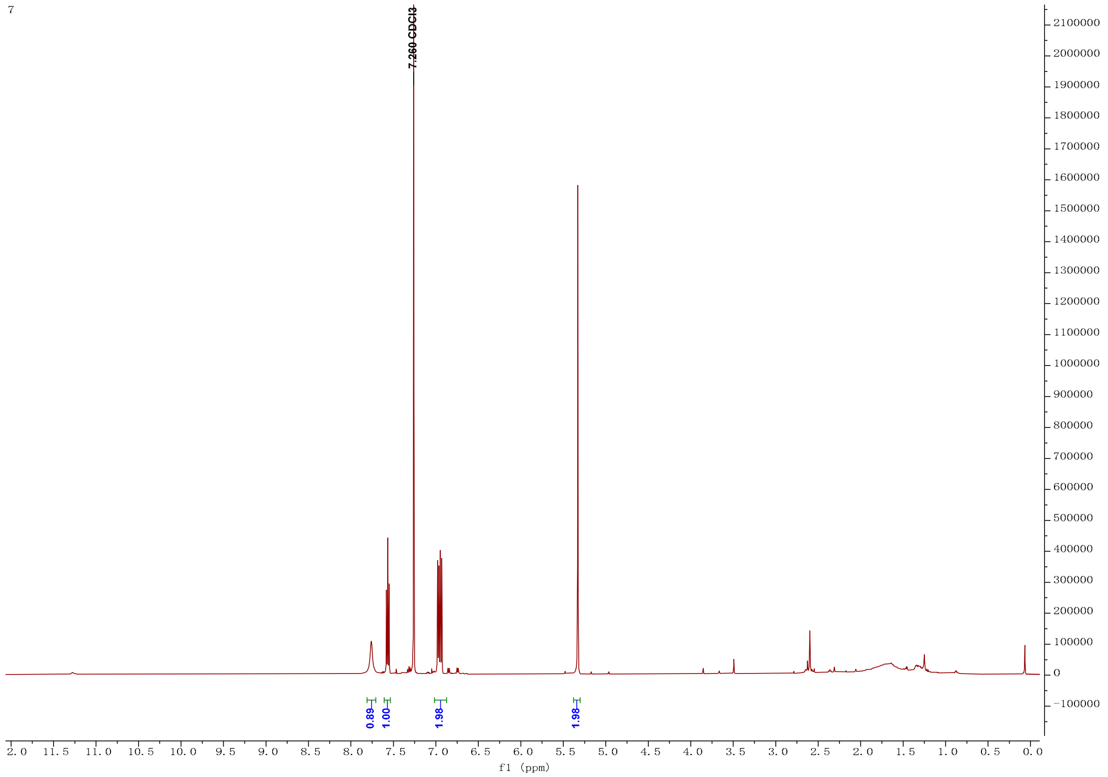

Figure S13. ^{1}H-NMR Spectrum of 7-Hydroxy-1(3H)-isobenzofuranone in $CDCl_3$

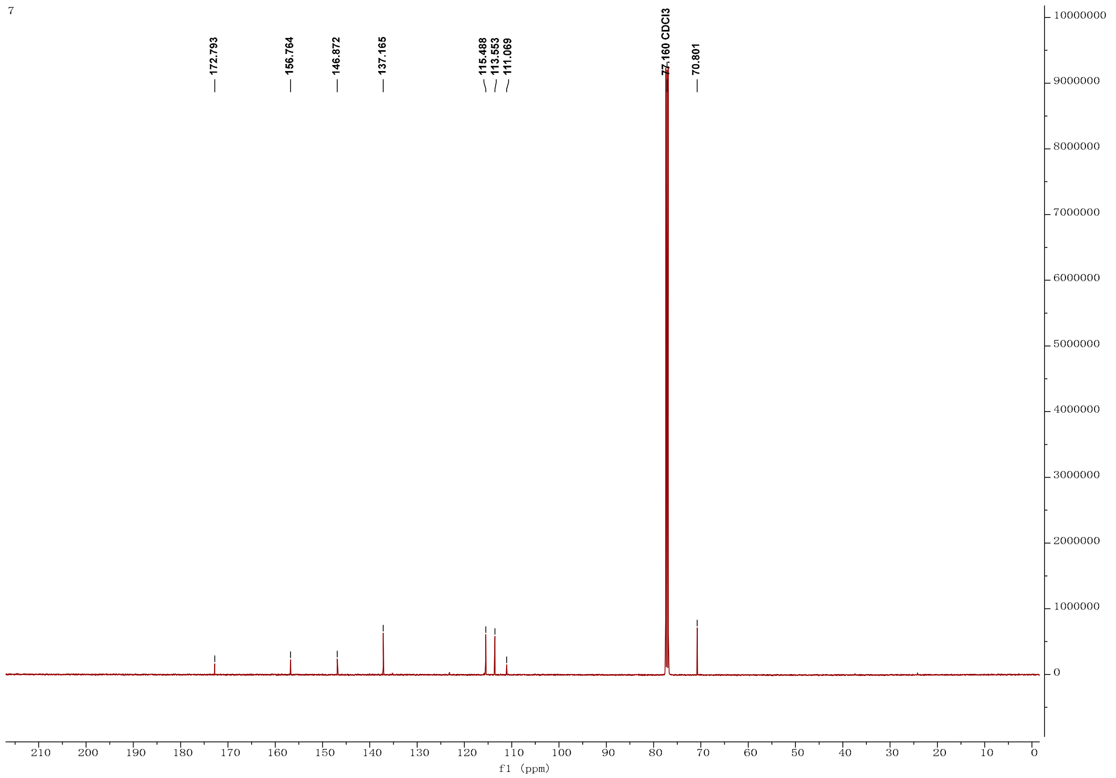

Figure S14. ^{13}C-NMR Spectrum of 7-Hydroxy-1(3H)-isobenzofuranone in $CDCl_3$
